# Postnatal gestational age estimation via newborn screening analysis: application and potential

**DOI:** 10.1080/14789450.2019.1654863

**Published:** 2019-08-17

**Authors:** Lindsay A. Wilson, Malia SQ. Murphy, Robin Ducharme, Kathryn Denize, Nafisa M. Jadavji, Beth Potter, Julian Little, Pranesh Chakraborty, Steven Hawken, Kumanan Wilson

**Affiliations:** a Clinical Epidemiology Program, Ottawa Hospital Research Institute, Ottawa, Canada; b Newborn Screening Ontario, Children’s Hospital of Eastern Ontario, Ottawa, Canada; c Department of Epidemiology and Community Health, University of Ottawa, Ottawa, Canada

**Keywords:** Gestational age, metabolomics, newborn screening

## Abstract

**Introduction**: Preterm birth is a major global health concern, contributing to 35% of all neonatal deaths in 2016. Given the importance of accurately ascertaining estimates of preterm birth and in light of current limitations in postnatal gestational age (GA) estimation, novel methods of estimating GA postnatally in the absence of prenatal ultrasound are needed. Previous work has demonstrated the potential for metabolomics to estimate GA by analyzing data captured through routine newborn screening.

**Areas covered**: Circulating analytes found in newborn blood samples vary by GA. Leveraging newborn screening and demographic data, our group developed an algorithm capable of estimating GA postnatally to within approximately 1 week of ultrasound-validated GA. Since then, we have built on the model by including additional analytes and validating the model’s performance through internal and external validation studies, and through implementation of the model internationally.

**Expert opinion**: Currently, using metabolomics to estimate GA postnatally holds considerable promise but is limited by issues of cost-effectiveness and resource access in low-income settings. Future work will focus on enhancing the precision of this approach while prioritizing point-of-care testing that is both accessible and acceptable to individuals in low-resource settings.

## 1. Introduction

Preterm birth, defined as birth that occurs before 37 weeks’ gestation, is a major public health concern worldwide, affecting nearly 15 million births in 2014 (10.6% of the total births [1]). Complications resulting from preterm birth are the leading cause of death among children under five, accounting for 35% of all global neonatal deaths in 2016 [1]. Infants who survive are at increased risk of a variety of complications, including cerebral palsy, sensory deficits, respiratory illness, and poorer cardiometabolic outcomes [2,3].

Rates of preterm birth vary considerably by geographic region, with 2014 estimates ranging from 8.7% in Europe to 13.4% in North Africa [3]. However, there is uncertainty as to the accuracy of these estimates in low- and middle-income settings due to the limited availability of prenatal ultrasounds and the unreliability of last menstrual period as an indicator of gestational age (GA) due to imperfect recall and documentation [4,5]. In addition, in many settings, preterm birth estimates are not reported or are not classified according to internationally accepted standards [6]. These limitations are significant, as accurate estimates of GA are important at both a population and individual level. Without comprehensive population-level data, appropriate resource allocation and program evaluation to support local and global health initiatives are impeded. At the individual level, accurate GA knowledge can help to direct care, particularly for term small-for-gestational-age (SGA) infants, who may appear similar to preterm infants in terms of size and birth weight. The ability to distinguish term SGA from preterm infants directly impacts both clinical decision-making and expectations for the achievement of developmental milestones.

In the absence of gold-standard prenatal ultrasound technology, several postnatal GA measurements exist, including the Ballard and Dubowitz scores, both of which are based on physical and neurological assessments of developmental milestones that occur in a predictable sequence over time [7–9]. A recent systematic review of GA assessment methods indicated that the Dubowitz score dates 95% of pregnancies to within ±2.6 weeks of ultrasound-estimated GA, while the Ballard score dates pregnancies to within ±3.8 weeks of ultrasound-estimated GA [10]. Another approach to postnatal GA estimation is through the evaluation of the newborn’s anterior lens capsule vascularity (ALCV); the disappearance of ALCV is a normal embryological process that occurs between 27- and 34-weeks’ gestation [11]. ALCV can be evaluated within 48 h of birth and is a good indicator of preterm birth (correlation: −0.719 [12]), as the vessels are usually completely resorbed by 35 weeks’ gestation [11]. This approach is highly dependent on environmental conditions, requires access to technology capable of producing high-quality images, and is of limited utility in identifying late preterm infants with a gestational age above 35 weeks [12]. The low level of invasiveness and low cost of these measures are important considerations for implementation in low-income settings, but these methods are also subject to a high level of inter-user variability, are affected by factors such as ethnicity and geography, and have limited accuracy, particularly among preterm and SGA infants [7–10].

Given the importance of accurately ascertaining estimates of preterm birth and in light of current limitations in postnatal GA estimation, novel methods of estimating GA postnatally in the absence of prenatal ultrasound are urgently required. Previous and ongoing work conducted by our research group and others has demonstrated the potential for metabolomics to fill this gap. Metabolomics refers to the use of liquid chromatography and mass spectrometry for the ‘quantitative cataloging’ of metabolites in a biological sample [13]. Concentrations of metabolites in such samples may reflect underlying biological processes that are associated with fetal maturation and thus may correlate with gestational age. Newborn screening programs in many countries already use biological samples collected during the neonatal period to measure a range of metabolites. Thus, we sought to explore the potential for newborn screening-based metabolomics to offer additional insights into rates of preterm birth.

## 2. Associations between gestational age and circulating analytes in newborns

Newborn screening programs are public health initiatives intended to identify infants at risk of rare, treatable diseases that do not typically show symptoms during the neonatal period. Newborn screening programs in high-income settings may screen infants for as many as 50 different conditions [14]. Screening usually involves collecting a small sample of blood from an infant’s heel during the first few days of life [15]. Tandem mass spectrometry (MS/MS) is used for expanded newborn screening programs that target metabolic diseases via the analysis of amino acids and acylcarnitines. Other analytes, including 17-hydroxyprogesterone (17-OHP) and thyroid-stimulating hormone (TSH) for screening of congenital adrenal hyperplasia and congenital hypothyroidism, respectively, are typically analyzed by immunoassay. Hemoglobin analysis for sickle cell disease and other hemoglobinopathies is facilitated by isoelectric focusing or high-performance liquid chromatography (HPLC), and polymerase chain reaction may be used to confirm genetic abnormalities.

The circulating metabolites measured during newborn screening are known to be affected by GA, a factor that is considered in the interpretation of newborn screening results [16]. Amino acids including arginine, leucine, and valine differ by as much as 50% between extremely preterm and term infants [17]. These variations may reflect higher levels of catabolism or delays in hepatic maturation among preterm infants. Newborn 17-OHP and TSH levels are also significantly correlated with GA at birth [17]. Levels of 17-OHP increase with increasing levels of prematurity, potentially due to heightened levels of neonatal stress. By contrast, levels of TSH can be as much as 60% lower among preterm infants compared to term infants, likely as a result of decreased thyroid and pituitary development [17].

In Ontario, Canada, nearly every infant born in the province undergoes newborn screening, and the results of this screening, including the individual analyte levels and key demographic variables, are stored by Newborn Screening Ontario (NSO), the provincial newborn screening program, until the child’s 19^th^ birthday.

Leveraging 2 years of linked health administrative and newborn screening analyte data from NSO for over 250,000 infants, our group developed an algorithm capable of accurately estimating GA to within 1 week of ultrasound-validated GA [18]. Model performance was evaluated across multiple birth categories: ≥37, 33–36, 28–32, ≤27 weeks’ gestation, and ≤34 and <37 weeks’ GA. Ryckman and colleagues at the University of Iowa and Jeliffe-Pawlowski and colleagues at the University of California, San Francisco used similar approaches to develop models capable of differentiating preterm from term births using data derived from 230,013 infants and 729,503 infants, respectively [19,20]. A summary of these approaches and their successes is provided in Table 1.

**Table 1.** Summary of gestational age modeling approaches.

Group	Ontario, Canada	Iowa, USA	California, USA
Newborn Screening Program	Newborn Screening Ontario	Iowa Newborn Screening Program	California Newborn Screening program
Sample size used for model development and testing	249,700	230,013	729,503
Years captured	2009–2011	2004–2009	2005–2011
Standard used for gestational age comparison	LMP, fetal ultrasound or combination	LMP or fetal ultrasound	Fetal ultrasound, 11–20 weeks’ gestation
Number of metabolite terms used in final model	44	37	35
Clinical factors included in final model	Birth weight, sex	Final model included metabolite terms only. Performance of final model evaluated with each addition of: month/year of collection, age (hour) at collection, birth weight, sex	Birth weight, age (hour) at collection
Infants correctly classified within 1 week of true gestational age	66.8%	78%	78.3%*
Infants correctly classified within 2 weeks of true gestational age (%)	94.9%	95%	91.7%*

*model performance amongst preterm infants only, <37 weeks’ gestation where 99.5% of the infants were correctly classified as ‘term’ or ‘preterm’. LMP: Last Menstrual Period

## 3. Model refinement and ethnic validation

After determining the functionality of this approach, it was important to assess the validity of the algorithm in different ethnic populations as a first step to determining its applicability in other countries. Birth weight, a significant predictor in all of our GA estimation models, is strongly correlated with GA and varies considerably by ethnicity; infants of European descent tend to have larger birth weights than other infants [21]. Infants of East Asian descent, who have a lower mean birth weight than other infants, are prone to misclassification as SGA when born in Western countries and assessed against growth curves derived in Western populations [22,23]. As our original GA estimation model was based on data from a sample of predominantly white infants and included birth weight among its model predictors, we conducted a retrospective validation study using data from infants in Ontario whose mothers were recent landed immigrants and compared model performance to that of infants born to mothers not identified as landed immigrants. In this way, we sought to determine whether one global algorithm would be adequate for estimating GA across ethnic subgroups, or whether local model calibration would likely be required in each new setting. Our results indicated that tailored algorithms may help to improve the precision of GA estimation, but our model performed well among infants from a variety of ethnic backgrounds [24], although there was some variation in accuracy of GA estimation. Among non-immigrant mothers, the model estimated GA to within an average of 1.05 weeks of true GA, while among immigrant mothers, estimates ranged from 0.98 to 1.15 weeks of true GA [24]. This suggested that our global model could perform well across a wide variety of settings but might be further improved through local calibration in new settings.

Recognizing that establishing new routine newborn screening programs in low-income settings may be hindered by technological and resource requirements, we also sought to refine the model to facilitate its implementation in these settings. Because of the known relationship between the ratio of fetal-to-adult hemoglobin (Hb) levels and GA [25], and the relative ease of measuring Hb levels compared to other newborn screening analytes (measured by HPLC versus MS/MS), we investigated the ratio of fetal-to-adult Hb as a potential new predictor of GA in our original Ontario sample. Though insufficient to predict GA on its own, the ratio of fetal-to-adult Hb in combination with clinical factors such as sex and birthweight estimated GA better than clinical covariates alone and improved upon the performance of our original Ontario-based algorithm described above [26]. Notably, Hb levels are relatively consistent and stable between blood spot samples taken via cord blood and heel-prick [27]. This differs from other analytes that fluctuate in the first few days after birth and may be inconsistent between cord and heel-prick blood spot samples due to differences in timing of collection. This consistency also has important implications in low-resource settings where there may be parental and/or health-care provider hesitancy surrounding heel-prick procedures, particularly among preterm infants [27].

## 4. International implementation

Having demonstrated proof-of-principle for metabolic estimation of GA, the effectiveness and practicality of using this approach in low-resource settings needed to be established. In 2016, we embarked upon a prospective validation study in Matlab, Bangladesh, nested within an existing preterm birth cohort established by the Global Alliance to Prevent Prematurity and Stillbirth (GAPPS). Health-care providers collected paired dried heel-prick and cord blood samples from the newborns of consenting mothers and gathered demographic data from the mother-child pairs. The dried blood spot samples were then shipped to Ottawa, Canada for analysis at NSO.

A total of 1,036 cord blood and 487 heel-prick samples were collected from 1,069 unique newborns. Collecting both heel-prick and cord blood samples enabled both validation of the model overall and evaluation of its relative performance in both sample types. When applied to heel-prick data, our algorithms estimated GA to within 1.07 weeks of ultrasound-validated GA overall, and correctly estimated GA to within 2 weeks for 94% of the infants. While model performance was slightly reduced when applied to data derived from cord blood samples, GA was correctly estimated to within 2 weeks for over 90% of the infants. These findings are encouraging, as cord blood sampling was more widely accepted by parents than heel-prick sampling due to concerns around causing discomfort to the infant or lack of understanding of the procedure. Health-care providers also reported being more comfortable collecting cord blood samples. The increased acceptability and uptake of cord blood sampling are an important consideration going forward if metabolic gestational aging approaches are to be scaled up to other settings. Importantly, the model performed especially well among infants whose birthweight was <2,500 g, among whom the use of the algorithm demonstrated the greatest improvement in GA estimation accuracy over estimation based on clinical information alone (i.e., sex, birthweight, multiple gestation), improving from a root mean square error (RMSE) of 2.21 to 1.44 [27]. This is of particular significance given the documented limitations of other postnatal GA estimation methods among SGA and low birthweight infants.

Our approach is now being implemented in real-world settings in sub-Saharan Africa and South-East Asia, in populations where prenatal care and use of gestational-dating ultrasounds are not widespread. In partnership with investigators at Stanford University, this initiative will result in prospective collection of heel-prick and cord blood samples from infants in Kenya, Zambia, Zimbabwe, and Bangladesh, permitting us to further define the accuracy of our algorithms in both types of samples, with a particular emphasis on cord blood.

## 5. Conclusion

It is well established that a difference in GA at birth of as little as 1 week can have significant impacts on neonatal morbidity, mortality, and long-term outcomes [28,29]. A problem in current clinical approaches to preterm infants is the limited availability of postnatal GA assessment tools that are both non-invasive and accurate, particularly among preterm and SGA infants. Evidence suggests that metabolomics techniques may be used to generate estimates of GA that are accurate to within 1–2 weeks of ultrasound-derived GA [18–20].

## 6. Expert opinion

Our modeling approach has evolved considerably over time and has now expanded to include advanced machine learning techniques to better accommodate large numbers of predictors relative to sample size, to incorporate flexible non-linear modeling of predictor-outcome associations, and to incorporate interaction effects among predictors and outcome. In order to better tailor our models as they are deployed in each new setting, we are developing model calibration and updating strategies to optimize model performance for local conditions. Calibration and updating in this way are yielding dramatic improvements in the accuracy of prediction and estimation.

We are also exploring state-of-the-art artificial intelligence (AI) modeling approaches such as deep learning neural networks (DLNN). The most advanced methods can handle much larger datasets with many more predictors and can identify and exploit more complex features in pursuit of highly accurate models, but these benefits come at the cost of more challenging interpretation and resource intensiveness. Although promising, these more advanced prediction and estimation models may provide diminishing returns compared to our latest conventional models with machine learning enhancements.

We are also undertaking studies that will examine the potential for an untargeted metabolomic approach to improve our ability to estimate GA postnatally and to identify infants at risk for a variety of conditions. The metabolites currently included in our GA model are restricted to those traditionally obtained through newborn screening and require further exploration to better elucidate their relationship to GA. The use of a broader spectrum of analytes may increase the accuracy of our model.

We continue to strive to enhance our approach to improve its feasibility and acceptability. A major challenge for the metabolomics approach to postnatal GA dating is the requisite use and expertise of advanced laboratory technology and resources to analyze biological samples. Previous work has demonstrated the feasibility of international testing of newborn samples, but such approaches are not economically sustainable in the long term [27]. For instance, in our Bangladesh cohort, the cost for shipment and analysis of each sample was approximately USD$50.00, excluding project start-up and maintenance costs. To address this, we have explored and continue to develop partnerships with established newborn screening programs in closer geographic proximity to sampling sites. We are also exploring a tiered approach to GA estimation that is based on birthweight and adapted to the region in which it is being implemented [21]. This approach would assume that infants above a certain weight threshold are term and would not require further testing. Below a certain weight threshold, infants could be classified as ‘potentially preterm’ and GA could be estimated using existing postnatal GA estimation measures, such as Anterior Lens Capsule Vascularity measurement while those between thresholds could undergo metabolomic GA dating. Additionally, adaptation of our model for reliable use on cord blood-derived data will likely enhance the acceptability of sample collection methods by family members and health-care providers who have expressed reticence about heel-prick sampling. By addressing these challenges, we may see this approach transition from a method of population-level surveillance to a tool that could be used to guide care for individual infants.

## Article Highlights

- Preterm birth is a major global health concern, contributing to 35% of all neonatal deaths in 2016. Complications resulting from preterm birth are the leading cause of death among children under five, accounting for 35% of all global neonatal deaths in 2016.
- The circulating metabolites measured during newborn screening are known to be affected by gestational age, and many of these metabolites are measured during routine newborn screening. Thus, we sought to explore the potential for newborn screening-based metabolomics to offer additional insights into rates of preterm birth.
- Leveraging 2 years of health administrative and newborn screening analyte data from NSO for approximately 250,000 infants, our group developed an algorithm capable of accurately estimating GA to within 1.06 weeks of ultrasound-validated GA.
- We then explored the addition of fetal-to-adult hemoglobin ratios into our model, and validated model performance among infants of immigrant mothers in Canada and a cohort of infants in Matlab, Bangladesh.
- Our ongoing efforts to improve the precision of our models in a variety of settings have demonstrated that metabolomics techniques may be used to generate estimates of GA that are accurate to within 1–2 weeks of ultrasound-derived GA. Future enhancements will be based on applying novel machine learning techniques to improve the accuracy of our models, and developing point of care testing to improve the intervention’s cost-effectiveness.
